# Supplementary material for: Outcomes of a primary care mental health implementation program in rural Rwanda: A quasi-experimental implementation-effectiveness study
Source: PLoS One. 2020 Feb 21;15(2):e0228854. doi: 10.1371/journal.pone.0228854 (PMC7035003; doi:10.1371/journal.pone.0228854)
Supplement: S1 File — (PDF) [file pone.0228854.s001.pdf]

Title

Measuring Service Use, Quality of Care and Outcomes of a Primary Care Mental Health Integration project in Burera district, Rwanda: a mixed-methods outcomes evaluation

Principle Investigators

Principal Investigator 1: Stephanie L. Smith, MD
Department of Global Health and Social Medicine
Harvard Medical School
United States
Email: stephanie_smith@hms.harvard.edu

Principal Investigator 2: C. Nancy Misago
Rwanda Biomedical Center
Rwanda

Co-Investigators

1. Hildegarde Mukasakindi, BA, Inshuti Mu Buzima

2. Beatha Nyirandagijimana, BA, Inshuti Mu Buzima

3. Manzi Anatole, MPH, Inshuti Mu Buzima

4. Dominique Dushimiyimana, Butaro Hospital, Rwanda Ministry of Health

5. Eugenie Uwimana, BA, Butaro Hospital, Rwanda Ministry of Health

6. Robyn Osrow, MD, Partners In Health/Inshuti Mu Buzima.

7. Molly Franke, SciD, Department of Global Health and Social Medicine, Harvard Medical School

8. Annie Michaelis, PhD, Partners In Health

9. Giuseppe Raviola, MD, MPH, Partners In Health, Department of Global Health and Social Medicine, Harvard Medical School.

Executive Summary

Background: A comprehensive evaluation of the IMB Mental Health program's innovative model of integrated mental health care at health centers (MESH MH) is needed to provide feedback for ongoing implementation and growth of the program. Evidence of increased uptake and acceptable quality of care by health center nurses, as well as improved patient clinical outcomes, must be documented before scale up of the MESH MH program to a national level could be considered.

Objectives/Methods: A mixed-methods outcomes evaluation with a pre- and post-test design will be performed at four purposively selected health facilities participating in the MESH MH program. Qualitative and quantitative outcome evaluations will be conducted to determine whether patients who receive mental health care at supported health centers (HCs) experience clinical and functional improvement and to explore the perspectives and experiences of health workers and patients who receive care through this program. As an adjunct to these evaluations, a process evaluation will be conducted using service utilization data to assess changes in uptake of mental health services at participating health facilities within the district, and use MESH supervision checklists to determine whether participating HC nurses adequately provide basic quality mental health care.

Population: Routine program monitoring data will be collected from the registries for all patients attending mental health services at district health centers and outpatient mental health clinic. All adults arriving for a mental health visit at selected health centers who are diagnosed with a neuropsychiatric disorder will be asked to participate in the quantitative portion of the evaluation. Patients who need to be transferred to the district hospital for an acute medical or psychiatric emergency and patients with a primary alcohol or substance use disorder will be excluded from the evaluation. Approximately eight nurses and fifty "information rich" patient and family participants will be selected purposively for the qualitative evaluation.

Outcome measures: Patients' mental health status, daily functioning, and economic status will be measured at baseline, eight weeks and four months using three clinician administered rating scales: the General Health Questionnaire (GHQ-12), the World Health Organization Disability Assessment Scale (WHO-DAS 2.0 Brief), and a brief economic questionnaire. Qualitative semi-structured interviews will be used to understand the meaning that users, families and providers ascribe to any statistical associations found in the quantitative analyses, and to provide rich descriptions of experiences and perceptions of the program.

Background and Rationale

Neuropsychiatric disorders account for about one quarter of the global burden of disability, yet rarely receive priority among the many health conditions competing for political will and allocation of funds. In most countries, the extreme shortage of mental health specialists has led to a gap of up to 90% between treatment need and available mental health services, despite the known efficacy of both pharmacological and psychosocial interventions for treating major mental disorders.[1 2] Innovative solutions that integrate mental health care into routine health platforms and optimize health worker interventions through 'task-shifting'– delegating tasks and responsibilities from more specialized to less specialized health workers – could contribute to effective treatment implementation and alleviating the burden of neuropsychiatric disorders in low income settings.[3 4 5 6 7]

The Republic of Rwanda's National Strategic Plan for Mental Health (revised 2011) calls to reduce the treatment gap in Rwanda by locating mental health services within public district health systems. Since 2005, the Ministry of Health (MOH)'s mental health decentralization efforts have succeeded in placing at least one psychiatric nurse and psychologist at each of forty one district hospitals in Rwanda,[8] although the ratio of public mental health workers to population within each district remains low. To address this shortage, the Strategic Plan also calls for the integration of mental health into primary care health services offered at health centers.[9] Evidence suggests that primary care workers who receive adequate training and supervision can successfully recognize and treat a range of mental disorders using evidence based practices.[10] Yet few models exist for the effective implementation and delivery of quality mental health services within primary care settings in resource limited areas.

Integration of mental health care into primary care is most likely to work well using existing systems of management and supervision as a starting point.[11 12] Partners In Health (PIH), a non-profit organization working in twelve countries, has been supporting the public health delivery system in three rural districts of Rwanda since 2005. In collaboration with the MOH, PIH/IMB (Inshuti Mu Buzima) has implemented the MESH program (Mentoring and Enhanced Supervision at Health Centers) to improve the quality of care provided by nurses at first level health facilities in IMB supported districts of Rwanda.[13] MESH supports health center nurse implementation of key child and adult clinical protocols by incorporating decentralized didactic training with ongoing clinical mentorship and quality improvement activities at health centers. The model follows closely with World Health Organization (WHO) clinical mentoring guidelines developed for effective task shifting of HIV care.[14] Initial results from the MESH program demonstrate significant improvement in a number of quality-of-care indicators after mentoring.[15] The MESH program is currently being brought to scale nationally for HIV care in all forty one districts in Rwanda, with preliminary plans in place for further future scale up in other clinical domains.

MESH Mental Health Program

MESH provided a robust platform through which to translate the national policy of decentralized mental health care into action and create a scalable model for primary care mental health services in rural Rwanda. In August 2012, IMB partnered with the MOH in Burera district, northern Rwanda, to integrate mental health care into health center primary care services using the MESH platform. Burera district is served by Butaro hospital, a 150-bed public hospital with approximately thirty five general nurses, thirteen full time general practitioners, four psychiatric nurses and one psychologist. The hospital is the primary referral center for seventeen health centers, and serves an overall population of approximately 340,000 people.

Three health centers in Burera district were initially chosen to pilot the MESH mental health (MESH MH) program. Four major neuropsychiatric disorders were chosen for clinical focus based on needs perceived by district mental health staff and health center directors, informal observation of diagnoses as listed in health center registers, and evidence of disease burden and treatment effectiveness as defined by the World Health Organization (WHO): schizophrenia, bipolar disorder, major depressive disorder, and epilepsy. After an intensive, forty-hour training designed to teach primary care nurses how to identify and manage these four disorders, a program of regular supervision by a trained Rwandan mental health nurse was initiated. Health center supervisory visits included clinical observation, individual case review, documentation review, and brief didactic sessions. A mental health supervision checklist was adapted from other MESH areas of clinical focus, to assist with clinical mentoring and to ensure standardization of activities across clinical domains. The mentor nurse used the case observation checklist to ensure that health center nurses were performing key elements of basic psychiatric evaluations, accurately diagnosing patients and offering appropriate treatment and support based on diagnosis. The successful completion of checklist items during mental health clinical evaluations was used to define basic quality of care provided by health center nurses. During each supervision session, the nurse-mentor also discussed systems based performance issues and "quality gaps" with the health center director and the health center nurses, and formulated specific plans to improve patient care, referral pathways, coordination between services, and other systems level issues.

Program Research Evaluation

Preliminary informal reports from Butaro district hospital staff, participating health center nurses and patients indicate that the MESH MH program has been received well at the three pilot health centers. In August 2013, the program was implemented at three additional health centers in Burera district, and in FY15 through FY17, the program will be implemented at all remaining health centers in the district. Although the program has an initial high level of acceptability and feasibility within the district health services, there is an urgent need for a comprehensive evaluation of MESH MH programming within the district system of care. Rigorous evaluation of MESH MH will provide valuable feedback for ongoing implementation and growth of the program. Additionally, evidence of increased service uptake and acceptable quality of care by

health center nurses, as well as improved patient clinical outcomes, must also be documented before scale up of the MESH MH program to a national level could be considered.

Study goals and objectives

Aim 1: Assess changes in uptake of mental health care by assessing the quantity and quality of mental health diagnoses at Burera district health centers in relation to the implementation of the MESH MH program.

Aim 2: Assess whether participating non-specialist health center nurses offer basic quality mental health care as specified in MESH MH program objectives.

Aim 3: Assess whether patients who receive mental health care at select health centers experience clinical and functional improvement, including economic status

Aim 4: Explore the perspectives and experiences of health center nurses, families and patients who receive care at select health centers where the MESH MH program has been implemented

Study Design

A mixed-methods outcomes evaluation with a pre- and post-test design will be performed. This design will allow for multiple data sources to be used to evaluate the MESH mental health program in its context. A simultaneous qualitative and quantitative outcome evaluation will be conducted to determine if the MESH MH program implementation is associated with clinical improvement for patients. A process evaluation will use service utilization data and supervision checklists (quality) to determine whether the MESH program has been successfully implemented at participating health centers and if the intended services are being provided. The process evaluation will serve as an adjunct to the outcomes evaluation in order to associate outcomes with rigor of implementation in program activities.

Methodology

Process Evaluation: routine quantitative program data

Routine program monitoring data will be collected for three months prior to and six months following the implementation of the MESH program, to assess whether increases in mental health diagnoses occur in relation to implementation of MESH. An increase in the number of visits following MESH implementation will be interpreted as an increase in uptake of mental health services.

All health centers participating in the MESH program in Burera district will be surveyed. At each health center, the total number of patients with a mental disorder seen at each health center per month, the number of new patients diagnosed with a mental disorder each month, and the number of patients who receive any follow-up over the course of six months will be recorded. In addition, demographics (including age, gender and village), and the diagnosis and treatment as

recorded in the health center register, will be documented for each patient. These routine data will also be collected from the Butaro district hospital outpatient mental health clinic to assess district wide service use.

Patient diagnoses and visit data are currently routinely recorded in the daily register by clinicians at all health facilities in the district. Each month, a research data officer will travel to participating health facilities and record these process indicators. The data will be entered into an electronic patient database currently in use by the IMB MH team for tracking routine process indicators. A subset of these data will be routinely reviewed for accuracy and completeness by the PI by comparing them with the paper registers at the participating facilities. The database is held on a team computer which is password protected and kept by the IMB MH team at Butaro hospital.

Data analysis: We will compare the number of new patients diagnosed with a mental disorder, the number of new mental health diagnoses, the number of mental health follow-up visits, and % of patients with specific diagnoses pre- and post- MESH MH program implementation. We will also compare new and follow up data from health centers with routine data from the district mental health clinic. We hypothesize that we will observe an increase in the number of mental health diagnoses and follow-up visits at health centers post-MESH implementation, representing an increase in uptake of mental health services and recognition of mental health morbidity among providers. We further hypothesize that MESH training will lead to improvements in the number of individuals with specific mental health diagnoses post-MESH.

Process Evaluation: routine quantitative quality of care data

Specific indicators of quality have been developed to track each nurse's provision of mental health care at health centers as an integral routine part of the MESH program. These quality of care indicators will be routinely collected over one year using the MESH supervision checklist, completed by mentors. Each month, all MESH mentor observation checklists are collected from the MESH nurse mentor and entered into a database. The quantitative process indicators for purposes of this evaluation will include a subset of routinely collected indicators and will include: the number of MESH checklists completed each week (indicating the number of directly observed cases), as well as the results from six dichotomous and two summed data points from each supervision checklist:

For new patients:
1) Did the nurse make a diagnosis?
2) Did the mentor agree with the diagnosis?
3) Safety questions
4) Summary score of treatment planning- non medication
5) Summary score of treatment planning- medication management
6) Did the nurse make a follow-up appointment?

Data analysis: For each nurse mentee from each health center, we will calculate the change in checklist score each month relative to baseline and test whether the mean change is different than zero. We hypothesize that within person change will increase with time since MESH implementation. For binary variables, we will examine whether proportions increase with time since MESH enrollment. We will adjust for correlation resulting from repeated measures from the same individuals.

Quantitative outcomes evaluation:

Study Population/Recruitment: All consecutive adults presenting to the mental health clinics at the four selected health centers for an initial visit between approximately October 2014 and January 2015, who have been diagnosed with a major mental or neurologic disorder, will be invited to participate in the quantitative outcomes evaluation. The mental health clinic day occurs once weekly as designated by the health center schedule. On that day, the clinician researcher will be available at the health center for enrollment in the research evaluation. Patients will be informed by the health center nurse, the MESH mentor and/or the clinician researcher present at the clinic, that a research evaluation of the mental health program will be occurring. If patients agree, the trained mental health clinician researcher will explain the evaluation further to the patient and determine whether the patient is willing to participate. Additionally, patients with a mental disorder who arrive at the clinic on a non-designated mental health clinic day will also be informed of the program evaluation by the health center nurse, and will be invited to return to participate the following week on the day that the clinician researcher will be present. Written informed assent from the patient and consent from his/her designated proxy will be obtained before data collection begins.

Data collection, measures and outcomes: The primary outcomes will be mental health status and daily functioning, measured at first visit, eight weeks and four months after beginning participation in the MESH MH program. Outcome measures will be determined by two clinician administered scales: the General Health Questionnaire (GHQ-12) and the World Health Organization Disability Assessment Scale (WHO-DAS 2.0 Brief). These scales have demonstrated high levels of validity and reliability and have been used extensively in developing countries. A brief economic status questionnaire for patients with mental disorders, adapted from other resource limited contexts, will also be administered.[16] All instruments will be translated into Kinyarwanda and back translated prior to implementation. The symptom and functioning scales will be used prior to the study by staff at the district mental health clinic and a small convenience sample of patients to ensure face validity.

The three scales will be administered by the trained clinician researcher who will be available on the designated mental health clinic days for questionnaire administration. If the clinician researcher determines that a patient is unable to offer adequate answers to questions secondary to severe mental illness (e.g. the patient exhibits clinical signs of severe psychosis such as disorganized thinking), the primary family member in attendance at the clinic with the patient

will be used as a proxy to answer questions, and the fact that a proxy has been used will be recorded. Demographic information as well as self-reported treatment status (new to treatment or received previous treatment) will be recorded. The PI will periodically check all completed questionnaires to ensure data quality and re-interview a random selection of respondents to verify data reliability.

Follow up: The mental health clinician researcher will maintain a list of patients who participated in the baseline data collection. Patients will be re-interviewed at eight week and four month follow up, on their return for follow up to the health center. For patients who do not return to follow-up, a community health worker in their village will be contacted to visit the patient and encourage the patient to return to care (as is routine practice in the current health system). Patients who return will be re-interviewed at eight week and four month follow up. The interviewer will not have sight of the previous questionnaires on follow up interviews.

Analysis: All participating patients will be included in the analysis. Patients who do not return for follow up will not be re-interviewed but will be included in final data analysis and we will examine baseline differences among those who do and do not complete follow-up interviews. Among patients who receive a score of > 2 on the WHO DAS-II (indicating non-zero baseline disability) and a score of >/= 3 on the GHQ-12 (indicating psychiatric caseness),[17 18] we will calculate the percent of patients who experience clinically significant reductions in score (25% for the GHQ-12 and 20% for WHO-DAS-II Brief). Since there is currently no information on the percentage of individuals anticipated to achieve these score reductions within our population, we will assume this to be 50% (the most statistically conservative for sample size calculations – we expect this percentage to be greater which will increase our precisions). A sample size of 96 patients will allow us to estimate these proportions and 95% confidence intervals with precision of +/-10. Assuming a drop-out rate of 20%, the minimum sample size for enrollment in the study will be 116. In order to stratify outcomes by characteristics such as age, gender, health center, diagnosis, and whether the patient is newly diagnosed or has previously been treated for a mental disorder, we will enroll as many patients are willing to participate (an estimate of 300 patients). We will use multivariable logistic regression to identify covariates associated with improved scores. Among individuals with low GHQ-12 and WHO-DAS-II Brief scores we will calculate within-person mean score change at eight weeks and four months. We will also calculate the percentage of patients who report improvements on the economic questionnaire and calculate mean score changes for individual responses.

Qualitative outcomes evaluation

Study Population/Recruitment: A subset of patients recruited for the quantitative program evaluation will be purposively selected for the qualitative program evaluation. Approximately 40-50 "information rich" (e.g. willing and able to speak freely about their experiences) participants will be chosen by the clinician researcher or recommended by the health center nurse or the MESH mentor. The interviews will occur between October 2014 and July 2015. Selected

patients and their accompanying family member will be invited to participate in the semi-structured interview by the clinician researcher at the second or third follow up for the quantitative evaluation.

Nurses: All health center nurses (approximately eight nurses) participating in the MESH MH program at the four selected health centers will be invited to participate in the qualitative program evaluation by the trained clinician researcher or the PI. The nurses will be invited sequentially to participate during the nine month period (October 2014 to May 2015) following the initiation of the MESH MH program.

Data Collection and Measurement: Demographic data of participants will be obtained. Semi-structured interviews will be conducted by the mental health clinician researcher who will be trained and supervised by the PIs. The interviews will be conducted in Kinyarwanda and audiotaped, and the interviewer will take notes for context and non-verbal communication. The interview guide will be developed through an emergent design including insights gained from the investigator's experience working as a psychiatrist in Burera district for two years, as well as insights from other mental health workers in Rwanda and the literature on primary care integration models for mental health.

The semi-structured interviews will include sections to discuss access to and uptake of care, quality of care and outcomes for patients of the MESH MH program, as well as the health center nurse as an agent of delivery and overall experiences of the MESH mentorship model of care, including perceived needs for improvement. Interview guides will be tailored to the nurses, users and families, probing for experiences and opinions in each section. Interviews will be translated into English and transcribed for analysis.

Data Analysis: A content analysis will be conducted. Each transcript will be analyzed and coded for the themes of access to and quality of mental health care at the health center level, the health center nurse as an agent of delivery and the mentorship model of mental health care. Illustrative examples of any associations found in the quantitative outcome evaluation will also be identified and synthesized.

Safety Considerations

The program evaluation will take place within the context of mental health service provision at health centers. Patients who are asked to participate are by design enrolled in follow up care at the health centers, and each participant will be provided follow-up care indefinitely both during and after the program evaluation is completed, as in the course of routine services.

The quantitative program evaluation consists of patient participation in a 15-30 min interview at baseline, eight weeks and four months documenting quantitative changes in clinical and functional status. Some patients and their family members will also participate in the qualitative interviews, which will involve participating in a 30-60 minute interview of open-ended questions

about mental health care at health centers. Health center nurses will also participate in these semi-structured interviews. Although answering questions about current clinical status and opinions about care provision is low risk for patients, families and nurses, there is a possibility that such discussions may cause an increase in distress for participants. To mitigate this risk, the research assistant will emphasize during the informed consent process that patients or family members can return to the health center for support should they experience such distress. Health Center nurses who may feel distress after participation will have access to support from the MESH mentor associated with their health center. If any acute safety risks are identified during or after the quantitative or qualitative interview processes (e.g. patient expression of an acute risk of harm to self or others), the participant will be referred to district mental health services at Butaro hospital for clinical evaluation.

Ethical Considerations

This proposal is for review by the Institutional Review Board of Harvard University and the Rwanda National Ethics Committee. The following consent process has been adapted according to the recommendations of RNEC. As patients with mental disorders are considered a vulnerable population; therefore, a more intensive consent process will be used to ensure that appropriate consent for participation in the study is obtained. Evidence does suggest that when systematic and thorough informed consent processes are implemented, patients with severe psychiatric disorders can understand and retain critical components of informed consent.[19]

Patients:

Quantitative Evaluation: The initial consent process will occur at the four selected health centers following patients' routine appointments for a mental disorder between approximately October 2014 and January 2015. In cases where patients are potentially interested in participating as determined by the recruitment procedure, the research assistant will describe the quantitative evaluation process in some detail, including the purpose of the study, the anticipated benefits and risks, and voluntariness issues, to both the patient and his/her accompanying family member, and answer any questions which may arise. If the patient and family member then agree to participate, the research assistant will then obtain assent from the patient and consent from the accompanying family member

The research assistant will be charged with obtaining assent and consent from patients and their family members, to reduce any sense of coercion to participate that may arise if a direct health care provider were responsible for obtaining consent. A strong emphasis will also be placed on how participation or non-participation will not affect care provided at the health centers, in order to mitigate any fear that non-participation will impact services received by patients. Patients will be reminded throughout the study period that their participation is voluntary and continuing assent to continue will be ascertained verbally at both follow up study visits.

Qualitative Evaluation: The initial consent process for the qualitative evaluation will occur at the four selected health centers following patients' routine appointments for a mental disorder between approximately December 2014 and September 2015. Purposively selected "information rich" patients who have assented to the quantitative evaluation will be asked with their family members by the research assistant if they are both willing to participate in a qualitative evaluation consisting of the longer semi-structured interview. The research assistant will then describe the qualitative evaluation process in some detail, including the purpose, anticipated risks and benefits, voluntariness and confidentiality issues. If the patient and family member agree to participate, the research assistant will then obtain assent from the patient and consent from the accompanying family member.

Nurses:

Qualitative Evaluation: The initial consent process for the nurses' participation in the qualitative program evaluation will occur between December 2014 and June 2015. The research assistant will obtain consent from nurses who have preliminarily agreed to participate during the initial recruitment process. The research assistant will describe the qualitative evaluation process in some detail, including the purpose, anticipated risks and benefits, voluntariness and confidentiality issues, and obtain written consent from all participating nurses

A separate form outlining the limits of confidentiality will also be discussed and signed by both patients and nurses.

Ethical issues:

1. As this program evaluation occurs within the course of service provision for patients with mental disorders, one ethical challenge will be mitigating any fear that that non-participation in the study will impact the services received by patients at the health center. This fear will be mitigated by strong emphasis in the informed consent process on how participation or non-participation will not affect patient's ability to obtain services at the health center. In addition, the research assistant will be charged with obtaining informed consent, to reduce any sense of coercion to participate that may arise if the patient's direct health care provider were responsible for obtaining consent.

2. Although the study takes place entirely within the context of service provision at health centers, some participating patients (e.g. patients without mutuelle) may find the follow up visits prohibitively expensive. If the research assistant becomes aware of participating patients who may qualify for transport and/or mutuelle assistance as specified in IMB MH program objectives, he/she will refer the patient to the Program Manager for Mental Health at IMB for further social assistance.

Confidentiality

To maintain participant confidentiality, all quantitative evaluation questionnaires will be completed in pen and paper format or on a password protected tablet and stored in a locked storage cabinet at Butaro hospital. Data will be entered from these questionnaires into a database on a password protected computer which is also stored in the locked cabinet. Qualitative interviews will be recorded and transcribed onto the same computer by the research assistant or certified translator. Audio recorders will also be held in the locked cabinet and erased after the study is completed. The PIs and co-collaborators will have sole access to these data during collection and analysis.

Limitations

There are limitations to the study design. First, this project endeavors to evaluate the plausibility that the MESH MH program contributes to improved clinical and functional outcomes among patients participating in the program, but it does not include a control group. The ability to draw causal inferences about the effectiveness of the MESH MH program in improving patient outcomes will therefore be limited. The use of program process indicators as an adjunct to the qualitative and quantitative outcome assessment will also help to ensure confidence that the MESH MH program has been implemented as intended at studied health centers. The addition of the process evaluation will strengthen the plausibility that any observed clinical changes could be attributed to the MESH MH program.

A second limitation is that the MESH mental health program is currently being implemented in public health centers in only one district in Rwanda which is well supported by Partners In Health (PIH). Thus, health system programming results may not be generalizable across all districts. For example, the in-country PIH psychiatrist will play an intensive role in supervision and monitoring of the program; this level of supervision may not be feasible in other districts and could influence the quality of delivery of the program, as well as patient outcomes. However, this plausibility evaluation will be the first report on service uptake, care quality and patient outcomes of a newly implemented supervised mental health program at the health center level, which may lead to further rigorous testing of the effectiveness of the intervention as it is scaled to other health centers and districts in Rwanda.

Expected Outcomes

Mental health has been envisaged as a key element of overall health and wellbeing since the 1978 Declaration of Alma-Ata,[20] and integrating mental health into primary care has since become a key strategy for reducing the burden of mental disorders for most low-income countries. Yet few studies exist to show that evidence-based mental health care can actually be effectively integrated and delivered in primary health care settings in resource-limited areas. The MESH MH program is the first systematic, new approach to integrated mental health care in low-income regions that capacitates front line health providers to successfully care for patients with severe mental disorders. A rigorous comprehensive mixed methods outcome evaluation to

determine whether the MESH MH program accomplishes that goal adequately will be among the first of its kind and will provide practical, instrumental information for ongoing IMB MH program decision making and improvement. In addition, an outcomes assessment which shows improved access to care and clinical outcomes for patients in rural areas of Rwanda would provide increased support for the MESH MH program as a scalable model of care. Robust evidence of an integrated mental health care model in a resource poor setting will provide the Ministry of Health with an important advocacy tool for obtaining both internal and external funding for mental health in Rwanda. Innovating rich mixed methodology for program evaluation, which includes both rigor and openness in examining the outcomes of the MESH MH program, ultimately will also provide more valuable and relevant information for national and international mental health system planners than what currently exists in the international literature.

Dissemination of Results and Publication Policy

Quantitative and qualitative data from this program will improve understanding of how the MESH MH program impacts access to, and quality of, mental health care at participating health centers in Burera district, as well as clinical outcomes for patients receiving care at these health centers. If results are positive, recommendations for scale-up will be developed and presented to key community, government, and NGO stakeholders. This will be facilitated by the participation of C. Nancy Misago, Head of Community at the RBC Mental Health Desk in Rwanda, as a principle investigator on the project.

Results will also be disseminated through high impact peer-reviewed publications and dissemination events in Burera district, in Rwanda and in other parts of Africa. The dissemination of the primary evaluation outcomes for publication will be completed by the Primary Investigators on the project, but co-collaborators will also be offered the opportunity to participate in, or lead, dissemination of project results as opportunities arise.

Abbreviations

MESH: Mentoring and Enhanced Supervision at Health Centers

MH: Mental Health

IMB: Inshuti Mu Buzima

HC: Health Center

GHQ-12: General Health Questionnaire, 12 item

WHO-DAS Brief: World Health Organization Disability Assessment Scale, Brief version

MOH: Ministry of Health

PIH: Partners In Health

PI: Principal Investigator

RBC: Rwanda Biomedical Center

IRB: Insitutional Review Board

NHRC: National Health Research Council

RNEC: Rwanda National Ethics Committee

Appendices

Attached

References

[1] World Health Organization (WHO): Atlas: mental health resources in the world. Geneva: WHO; 2005

[2] Eaton JE, McKay L, Semrau M, et. al. Scale up of services for mental health in low-income and middle-income countries. *Lancet* 2011; 378 (9802), 1593-1603.

[3] Patel V, Belkin GS, Chockalingam A, Cooper J, Saxena S, Unützer J. Grand challenges: integrating mental health services into priority health care platforms. PLoS Med 2013; 10(5).

[4] Patel, et.al. Treatment and prevention of mental disorders in low-income and middle-income countries. *Lancet* 2007; 370 (9590), 859-877.

[5] Raviola G, Becker AE, Farmer P. A global scope for global health--including mental health. *Lancet* 2011; 378(9803):1613-5.

[6] Bass JK, Bornemann TH, Burkey M, Chehil S, Chen L, et al. A United Nations General Assembly special session for mental, neurological and substance use disorders: the time has come. *PLoS Med* 2012 9(1).

[7] Collins PY, Insel TR, Chockalingam A, Daar A, Maddox YT. Grand challenges in global mental health: integration in research, policy, and practice. *PLoS Med* 2013; 10 (1)

[8] Kayiteshonga, et al. Building a national mental health system in Rwanda: Prioritizing equity, decentralization, and integration. In submission.

[9] Patel, et. al. Treatment and prevention of mental disorders in low-income and middle-income countries. *Lancet* 2007; 370 (9590), 859-877.

[10] World Health Organization. Integrating mental health into primary care: a global perspective. WHO, Geneva, 2008. Accessed 4/1/14. Available at: http://www.who.int/mental_health/policy/services/integratingmhintoprimarycare/en/index.html.

[11] Patel V, Belkin GS, Chockalingam A, Cooper J, Saxena S, Unützer J. Grand challenges: integrating mental health services into priority health care platforms. *PLoS Med* 2013; 10(5).

[12] Lund C, Tomlinson M, De Silva M, Fekadu A, Shidhaye R, et al. (2012) PRIME: A Programme to Reduce the Treatment Gap for Mental Disorders in Five Low- and Middle-Income Countries. *PLoS Med* 9(12).

[13] Anatole M, Magge H, Redditt V, Karamaga A, Niyonzima S, Drobac P, Mukherjee JS, Ntaganira J, Nyirazinyoye L, Hirschhorn LR. Nurse mentorship to improve the quality of health care delivery in rural Rwanda. *Nurs Outlook.* 2013; 61(3):137-44.

[14] World Health Organization. (2005). WHO Recommendations for Clinical Mentoring to Support Scale-up of HIV Care, Antiretroviral Therapy and Prevention in Resource-Constrained Settings. Geneva: WHO.

[15] Anatole M, Magge H, Redditt V, Karamaga A, Niyonzima S, Drobac P, Mukherjee JS, Ntaganira J, Nyirazinyoye L, Hirschhorn LR. Nurse mentorship to improve the quality of health care delivery in rural Rwanda. *Nurs Outlook.* 2013; 61(3):137-44.

[16] Lund C, Waruguru M, Kingori K, Kippen-Wood S, Breuer E, Mannarath S, Raja S. Outcomes of the mental health and development model in rural Kenya: A 2-year prospective cohort intervention study. International Health 2013; 5: 43-50.

[17] Andrews G, Kemp A, Sunderland M, Von Korff M, Ustun TB. Normative Data for the 12 Item WHO Disability Assessment Schedule 2.0. *PLoS ONE* 2009 4(12): e8343

[18] http://www.nwph.net/lifestylesurvey/userfiles/mental/things/GHQ12.pdf

[19] Wirshing DA, Wirshing WC, Marder SR, Liberman RP, Mintz J. Informed Consent: Assessment of Comprehension. *Am J Psychiatry* 1998;155:1508-1511.

[20] http://www.who.int/publications/almaata_declaration_en.pdf
