# Supplementary material for: Outcomes of a primary care mental health implementation program in rural Rwanda: A quasi-experimental implementation-effectiveness study
Source: PLoS One. 2020 Feb 21;15(2):e0228854. doi: 10.1371/journal.pone.0228854 (PMC7035003; doi:10.1371/journal.pone.0228854)
Supplement: Attachment — Submitted filename: Response to Reviewers.docx [file pone.0228854.s004.docx]

Responses to the Journal’s requirements (also addressed in the Cover Letter):

1) As a point of clarification, the organization Partners In Health is a non-profit 501(c)(3) organization, not a commercial entity as the editor mentions in point 1 of the ‘Journal Requirements’. We have edited our Funding Statement and Competing Interests statement as requested by the Editor, but want to make sure that our organization is not misrepresented as a commercial company.

Responses to the Editor’s comments:

1) We have added a section on how our sample size was determined (entitled Sample Size in the Methods section). We have also added into the Abstract a statement on the primary limitation of the study (lack of a control group), and have discussed this limitation and possible effects of the limitation in paragraph 8 of the Discussion section, particularly that the clinical improvements observed in the study may be due to underlying secular trends or the natural course of illness in our population rather than the initiation of program implementation.

Responses to Reviewer #1:

1) As requested, we have rewritten the abstract to distinguish clearly between methods and results. We have included a brief description of the key elements of the MESH MH implementation program within the methods section, as noted by the reviewer. In addition, we clarify that the population participating in the outcome evaluation is a group of consecutive adults presenting to the health centers over a nine month period—a subset of the total number of patients seen over the two year scale-up period. We have stated the primary limitation of the study in the conclusion of the abstract. Please also refer to the tracked changes version of the revised manuscript.
2) In comment #2, the reviewer asks for expansion of the discussion on the differences among checklist item completion subgroups (assessment versus intervention). These differences, and specifically the variable scores of the nurses on non-pharmacologic interventions, as mentioned by the reviewer, is discussed relatively extensively in paragraph 5 of the Discussion section. We have now made some minor edits to the section to make more clear that we are discussing the issues put forth by the reviewer. If additional expansion is still requested beyond our edits, please give further detail on what elements we should expand on given that this section of the Discussion is relatively lengthy already.
3) We have added a comment in the Results section highlighting the role of CHWs for those who continued in care after six months.

Responses to Reviewer #2:

1) In the Methods, in the section ‘Evidence-based Care Packages’, we have made clearer how the initial areas of clinical focus (psychosis, severe depression and epilepsy) were chosen. The specific clinical areas were determined both by perceived burden of illness attributable to these disorders within the district, as well by the stated clinical priorities of health system leaders in Burera district during the early stages of MESH MH development.
2) We agree with the reviewer that in many ways, the program was designed from a health system perspective in mind—namely that the program components were focused on improving quality of care delivered by non-specialist providers, and more could be added to the program around supported decision-making with service users. We have added some text in paragraph 8 of the Discussion section about next steps and need for program improvements vis a vis effective participation in program development by service users.
3) In the Results, in the section entitled ‘Evaluation of participants and follow-up’ we have clarified how many individuals (four) were excluded from the program evaluation due to acute psychiatric or medical needs.
